# Protein S in preventing thrombosis

**DOI:** 10.18632/aging.101798

**Published:** 2019-01-25

**Authors:** Doerte R. Fricke, Sabyasachi Chatterjee, Rinku Majumder

**Affiliations:** 1 Department of Biochemistry, LSU Health Science Center, New Orleans, LA 70112, USA

**Keywords:** protein S, factor IXa, activated protein C, fibrin and heparin binding exosite

Blood clotting is obviously important for wound healing but, if induced abnormally, clotting can cause thrombosis. Natural anticoagulants, such as antithrombin, activated protein C, tissue factor pathway inhibitor, and protein S (PS) are needed to prevent aberrant thrombus formation [1]. PS contains an N-terminal Gla domain, thrombin sensitive region (TSR), four epidermal growth factor (EGF)-like domains, and a sex hormone binding globule (SHBG) [2]. Heterozygous PS deficiencies are associated with increased risks of venous thromboembolism; homozygous PS deficiencies may cause the fatal thrombotic condition neonatal purpura fulminans. These facts illustrate the important function of PS in controlling blood coagulation [3].

In the regulation of blood coagulation, PS is a co-factor of APC and TFPI (4] [5]. Additionally, PS binds directly to, and inhibits factor IXa (FIXa) *in vitro* [6,7]. FIX is a key component in the regulation of clot formation and hemostasis. However, an elevated amount of FIXa increases the risk of venous thromboembolism [8]. Since elevation of FIXa causes thromboembolism, inhibition of FIXa will be a promising approach in the treatment of venous thromboembolism. Because PS has a vital function in the inhibition of FIXa, it is important to understand the mechanism of PS-dependent FIXa inhibition. This understanding will further advance the development of anti-thrombotic therapeutics.

To further understand how PS interacts with FIXa, binding of PS and FIXa to activated platelets was examined [8]. Co-localization of PS and FIXa was observed although, at this level, it was possible that the two proteins bound individually to the activated platelets. To determine whether co-localization was due to interaction between FIXa and PS, FIXa mutants were created lacking the membrane-binding Gla domain. Co-localization continued to be observed, which indicated direct protein-protein interaction between FIXa and PS and which did not require the FIXa Gla domain. Additionally, immunoprecipitation experiments revealed direct binding of FIXa and PS in plasma. Analysis of mouse thrombus formation in real time supported these findings by exhibiting co-localization of FIXa and PS *in vivo* [8].

In this study [8], an overlap was discovered between the heparin and PS binding sites in FIXa. Additionally, it was demonstrated that binding of PS to FIXa resulted in conformational changes in FIXa. To evaluate whether PS binds to the HBE in FIXa, FIXa mutants were created with amino acid substitutions in the HBE. Binding studies showed that the following FIXa mutants failed to bind PS *in vitro*: FIXa K126A, K132A, and K132A/R170A. However, FIXa K132A/R170A was the only mutant that retained full enzymatic activity. Therefore, this mutant was used for further studies described below. The binding studies confirmed the hypothesis that the HBE, particularly K132 and R170, in FIXa is important for PS binding; this conclusion was confirmed by experiments in the plasma environment [8]. The effects of select FIXa HBE mutations were further assessed by measuring PS-dependent inhibition of FX activation. PS binds both free FIXa and the intrinsic FX activation (Xase) complex as an allosteric inhibitor. However, PS was not able to bind several of the free FIXa mutants or the FIXa mutants incorporated into the intrinsic Xase complex; therefore, PS failed to inhibit FX activation.

Coagulation activity of FIXa WT, FIXa K132A/R170A, and FIXa R150A in FIX/PS double-immunodepleted plasma showed 90% decreased activity of FIXa WT and FIXa R150A mutant in the presence of PS. The FIXa K132A/R170A mutant, however, showed only 58% decreased activity in the presence of PS. This latter result indicated that the FIXa K132A/R170A mutant is resistant to PS binding in plasma. Evaluation of the behavior of the different mutants in thrombin generation in plasma strengthened this hypothesis [8].

*In vivo* studies with hemophilia B mice and the respective FIXa mutants showed increased fibrin accumulation in FIXa K132A/R170A mutant models compared to FIXa WT and FIXa R150A. This outcome supported the *in vitro* data that showed resistance of the FIXa K132A/R170A mutant to PS binding. Accumulation of fibrin in these models *in vivo* demonstrated the importance of the HBE domain in FIXa for PS-dependent inhibition [8].

**Figure 1:**
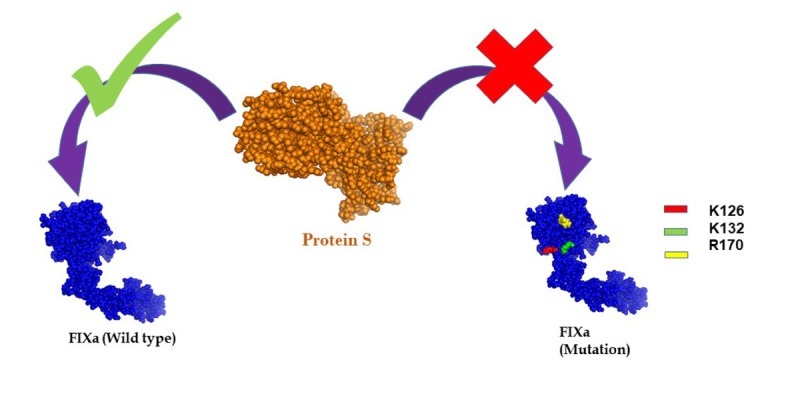
Wild type FIXa binds to Protein S, but the binding ability was abolished due to the mutation of the amino acid residues- lysine 126 (K126), lysine 132 (K126) and arginine 170 (R170) in the heparin-binding exosite region of FIXa.

In summary, direct binding of PS to FIX was confirmed *in vivo* and amino acid residues K126, K132, and R170 in FIX were identified as critical for PS binding (Figure 1). Most importantly, the FIXa K132A/R170A mutant maintained full activity but was unable to bind PS, a finding that confirmed the hypothesis that the HBE in FIXa is critical for PS-dependent inhibition of FIXa.. These results show the significance of PS-FIXa interaction in hemostasis and provide promising new therapeutic opportunities for thrombosis treatment by targeting the FIX exosite instead of the active site. This approach might decrease the risk of bleeding, which is an important negative side effect of anticoagulants.

Anticoagulant drugs are associated with a high risk of bleeding. Thus, there is a need for innovations in the treatment of thrombosis. Development of novel antithrombotic drugs requires advanced research in blood coagulation. Majumder et al. [8] recently generated a FIXa mutant with amino acid substitutions (K132A/R170A) in the heparin-binding exosite (HBE) and discovered that this exosite is necessary for protein S-dependent inhibition of FIXa *in vitro* and *in vivo*. The FIXa K132A/K170A mutant remained fully active but lost the ability to bind PS (measured *in vitro*). Additionally, hemophilia B mice injected with FIXa K132A/R170A showed increased fibrin accumulation compared with mice that received FIXa WT. This observation revealed a major function of the HBE in PS-dependent FIXa inhibition. Importantly, these results provide the basis for a promising new approach to antithrombotic therapeutics. By targeting the FIXa’s exosite instead of its active site, the risks of side effects caused by commonly used anticoagulants are expected to decrease dramatically. Therefore, the results of this study may have a major impact on future drug development.
